# Multi‐Model Ensembles in Ecosystem Modeling: Challenges and Best Practices for Decision‐Making

**DOI:** 10.1111/gcb.70987

**Published:** 2026-08-14

**Authors:** Kaiyu Guan, Rongzhu Qin, Zewei Ma, Lei Zhao, Licheng Liu, Sheng Wang, Trevor F. Keenan, Mengqi Jia, Debjani Sihi, Eric Potash, Senthold Asseng, Jingyun Tang, Xiangtao Xu, Katheleen Boomer, Bin Peng, Zhenong Jin, Forrest M. Hoffman, Stephen Ogle, Jonathan Coppess, Madhu Khanna, Alyssa Whitcraft, Benjamin R. K. Runkle

**Affiliations:** ^1^ Agroecosystem Sustainability Center, Institute for Sustainability, Energy, and Environment University of Illinois Urbana‐Champaign Urbana Illinois USA; ^2^ Department of Natural Resources and Environmental Sciences, College of Agricultural, Consumer and Environmental Sciences University of Illinois Urbana‐Champaign Urbana Illinois USA; ^3^ DOE Center for Advanced Bioenergy and Bioproducts Innovation University of Illinois Urbana−Champaign Urbana Illinois USA; ^4^ National Center for Supercomputing Applications University of Illinois Urbana‐Champaign Urbana Illinois USA; ^5^ NASA Acres Consortium Washington DC USA; ^6^ Department of Civil and Environmental Engineering University of Illinois Urbana‐Champaign Urbana Illinois USA; ^7^ Institute for Sustainability, Energy, and Environment (iSEE) University of Illinois Urbana‐Champaign Urbana Illinois USA; ^8^ College of Agricultural and Life Sciences University of Wisconsin–Madison Madison Wisconsin USA; ^9^ Department of Agroecology, Pioneer Center Land‐CRAFT University of Aarhus Aarhus Denmark; ^10^ Climate and Ecosystem Sciences Division Lawrence Berkeley National Laboratory Berkeley California USA; ^11^ Department of Environmental Science, Policy and Management University of California, Berkeley Berkeley California USA; ^12^ Department of Plant and Microbial Biology, N.C. Plant Sciences Initiative North Carolina State University Raleigh North Carolina USA; ^13^ Department of Crop and Soil Sciences, N.C. Plant Sciences Initiative North Carolina State University Raleigh North Carolina USA; ^14^ Department of Life Science Engineering, Digital Agriculture, HEF World Agricultural Systems Center Technical University of Munich Freising Germany; ^15^ Molecular Ecology and Biogeochemistry Department Lawrence Berkeley National Laboratory Berkeley California USA; ^16^ Department of Ecology and Evolutionary Biology Cornell University Ithaca New York USA; ^17^ Foundation for Food and Agricultural Research Washington DC USA; ^18^ Department of Crop Sciences, College of Agricultural, Consumer and Environmental Sciences University of Illinois Urbana‐Champaign Champaign Illinois USA; ^19^ Department of Bioproducts and Biosystems Engineering University of Minnesota‐Twin Cities Saint Paul Minnesota USA; ^20^ Integrated Computational Earth Sciences Group Oak Ridge National Laboratory Oak Ridge Tennessee USA; ^21^ Department of Ecosystem Science and Sustainability Colorado State University Fort Collins Colorado USA; ^22^ Natural Resource Ecology Laboratory Colorado State University Fort Collins Colorado USA; ^23^ Department of Agricultural & Consumer Economics University of Illinois Urbana‐Champaign Urbana Illinois USA; ^24^ Department of Geographical Sciences University of Maryland College Park Maryland USA; ^25^ Department of Biological and Agricultural Engineering University of Arkansas Fayetteville Arkansas USA

**Keywords:** ecosystem modeling, model development, model evaluation, model intercomparison projects, model uncertainty, multi‐model ensembles

## Abstract

Ecosystem models are increasingly central to the decision‐making for environmental policy, conservation planning, and climate‐related investments. Yet, the growing reliance on Multi‐Model Ensembles (MMEs) of ecosystem models by practitioners and policymakers, sometimes under tight timelines and imperfect information, has frequently outpaced the scientific rigor required to ensure ensemble reliability. Here, MMEs refer to approaches that combine targeted predictions from multiple models with the expectation of improving robustness and quantifying predictive uncertainty. Poorly designed MMEs may create a false sense of confidence and lead to suboptimal policy and market decisions. This perspective argues that robust decision‐making‐relevant MMEs must be grounded on two pillars: (1) rigorous Model Intercomparison Projects (MIPs), which identify inter‐model agreement and disagreement, characterize model uncertainties, and evaluate robustness with observationally based benchmarks—MIPs' diagnostic evaluation is so critical that it must be needed to drive MME's decision in model selection and weighting, especially when only a limited number of models available; and (2) co‐design by both stakeholders and scientists to ensure that scenarios, metrics and uncertainty requirements provide decision‐relevant information. Building upon the past success and lessons from the existing MIPs‐MMEs efforts (e.g., climate/Earth system/crop), we derived the theoretical basis for MMEs, addressed their specific challenges in ecosystem modeling, and highlighted proper consideration of model numbers and diversity, risk of model inter‐dependence, effective calibration of model parameters, possible overdue of some ecosystem model development, critical roles of open benchmark data across a wide range of conditions, and suggested use of Artificial Intelligence to support MIPs‐MMEs. We highlighted the under‐recognized opportunity for MIPs and MMEs to drive scientific progress and innovation through identifying better performing models, systematic benchmarking, feedback loops, and targeted model improvement. By following actionable best practice guidelines, MMEs can evolve from ad hoc aggregation of models into a trusted backbone of environmental policy and decision‐making.

## Graphical abstract

As ecosystem models increasingly inform environmental policy, markets, and climate‐related investments, this article highlights a critical but often overlooked issue: combining multiple models does not automatically make predictions more reliable. We show that trustworthy multi‐model ensembles are strengthened by rigorous model intercomparison, attention to shared biases and model dependence, and co‐design with decision‐makers. By connecting model evaluation, iterative improvement, and stakeholder‐relevant uncertainty, this article provides a framework for using ecosystem model ensembles more credibly in real‐world decisions.

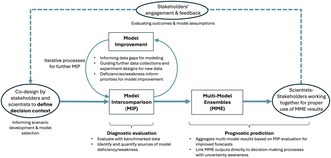

## 1 Introduction: Why MMEs Are Needed

Ecosystem models (Box 1) have become increasingly indispensable for policy and stakeholder decision‐making, given their ability to quantify complex and multi‐dimensional environmental impacts of different policy designs and industry activities (Anderegg et al. 2020). As environmental markets and conservation−/climate‐related finance rapidly expand, ecosystem models guide both public policy and important investment decisions for stakeholders ranging from policymakers, farmers, conservation groups, to ecosystem services market investors and more. Their outputs are increasingly employed in a variety of high‐stakes contexts, including national agricultural policies (e.g., U.S. Farm Bills, biofuel policies) (Coppess et al. 2025; 26 U.S.C. § 45Z; Inflation Reduction Act 2022), voluntary and compliance carbon markets (e.g., Article 6 of the Paris Agreement; Novick et al. 2024), and international and national assessment reports and policy design (such as IPCC 2023; Crimmins et al. 2023).

**FIGURE 1:**
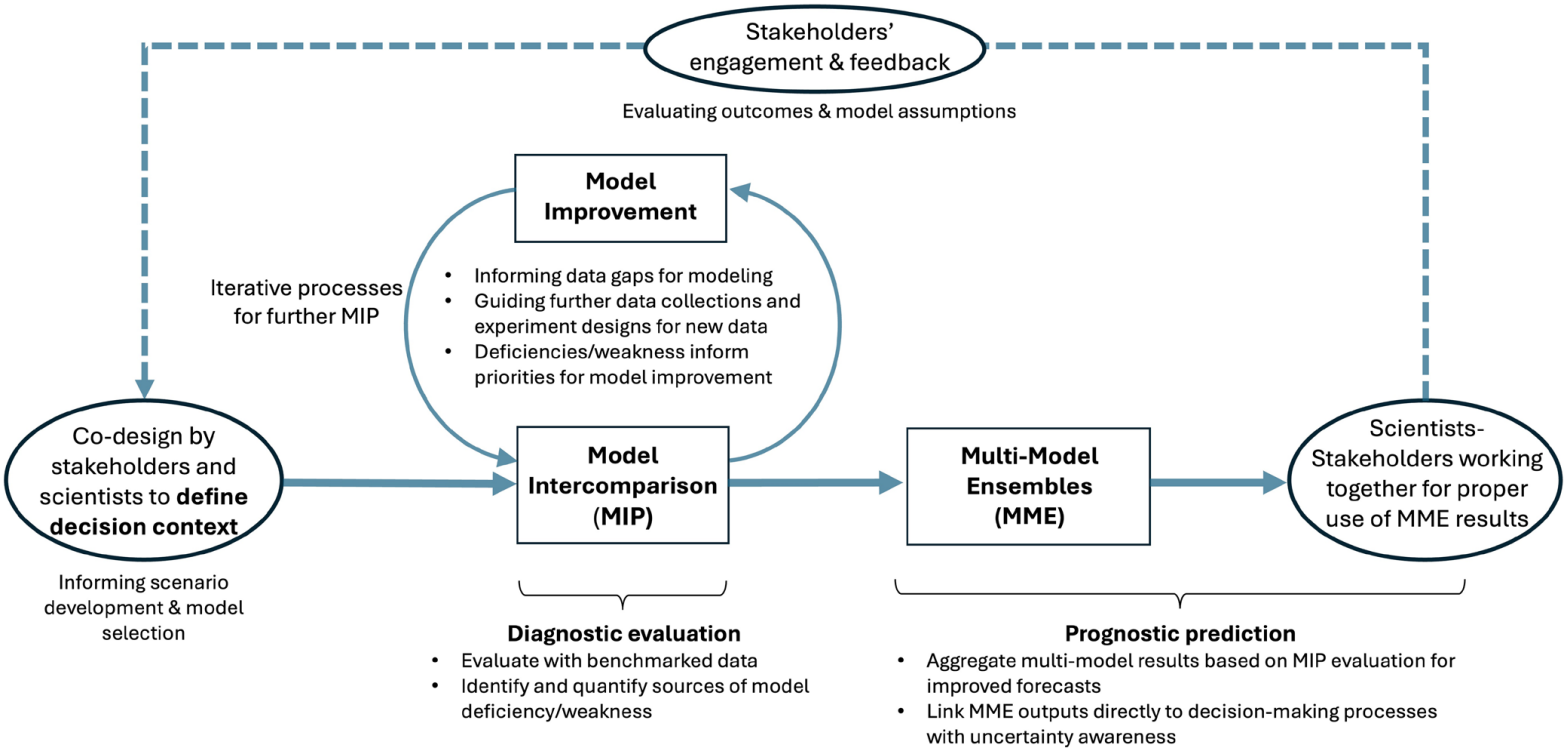
Conceptual diagram of proposed best practices for decision‐making‐relevant Multi‐Model Ensembles in ecosystem modeling. We highlight the important distinction of MIP as diagnostic evaluations, and MME as prognostic forecasting related to decision‐making.

Multi‐Model Ensembles (MMEs) (Box 1, Figure 1) have long been regarded as the recommended practice for climate, ecosystem, or crop modeling to synthesize insights from multiple models by aggregating their outputs to generate consensus‐based projections with robustness and uncertainty characterization. Integrating multi‐model results provides a compelling basis for quantifying uncertainty in model predictions, and thus strengthening public credibility (Leamer 1983; Layton and Lee 2006). Extensive empirical evidence shows that ensemble mean predictions frequently outperform individual models (Asseng et al. 2015; Martre et al. 2015; Simmons and Splinter 2022; Thomson et al. 2006; Wallach et al. 2018). In most cases, these improvements arise because averaging across models reduces model randomness (variance) and partially compensates for model‐specific biases (Hagedorn et al. 2005), rather than because all systematic errors are removed (see Section 2 for details). Thus, the performance of MMEs strongly depends on which models are included, how similar their errors are, and whether they share common biases. This issue is particularly important in ecosystem modeling, where the relatively small number of available models makes ensemble results especially sensitive to model choice, shared biases, and model inter‐dependence (see Section 3 for details). If MMEs are not carefully constructed and interpreted, this sensitivity of model selection can limit the usefulness of MMEs for policy and market decisions.

Practitioners supporting high‐stakes policy and investment decisions in complex, dynamic systems are increasingly considering MMEs to synthesize evidence and characterize uncertainty (e.g., Boehlert et al. 2015; den Boon et al. 2019; Scavia et al. 2017). The decision context to the specific decision being made and the conditions that shape it (e.g., goals and objectives, constraints, time horizon, and spatial scale) must be clarified to help ensure outputs are interpretable and actionable (Kolusu et al. 2021; Shea et al. 2020). MMEs tend to be most decision‐relevant when decision‐makers and stakeholders help define objectives, performance metrics, and scenarios, and when the ensemble design reflects stakeholder perspectives and key system drivers likely to influence outcomes at a scale relevant to the decision (Robinson et al. 2023; Sunkara et al. 2023; Townsend et al. 2014). When structured to compare stakeholder‐defined scenarios rather than producing a single “best” forecast, MMEs can help reveal key uncertainties, expose potential failure modes, and support adaptive risk management (Kolusu et al. 2021; Sunkara et al. 2023).

MMEs should not be treated as stand‐alone products, but rather as outcomes that must be firmly rooted in robust Model Intercomparison Projects (MIPs) (Box 1, Figure 1). While MMEs refer to the aggregation of predictions derived from multiple models, MIPs diagnose performance strengths and weaknesses of individual models, assess model diversity, and reveal sources of discrepancies across different model simulations benchmarked on observations. Existing notable MIP examples include Coupled Model Intercomparison Project (CMIP) for climate projection (Eyring et al. 2016), Agricultural Model Intercomparison and Improvement Project (AgMIP) (Rosenzweig et al. 2013), Trends and drivers of the regional scale terrestrial sources and sinks of carbon dioxide (TRENDY) (Sitch et al. 2024), North American Carbon Program Multi‐scale synthesis and Terrestrial Model Intercomparison Project (NACP MsTMIP) (Huntzinger et al. 2013), the Global N2O Model Intercomparison Project (NMIP) (Tian et al. 2018), etc. MIPs are uniquely positioned to quantify uncertainty relative to observations, a task that MMEs alone cannot accomplish. Therefore, MIPs could reduce the risk that MMEs reinforce due to potentially model‐shared structural biases or masked model deficiencies, by providing the foundational rigor for MMEs. In this sense, MMEs and MIPs should be viewed as tightly coupled and iterative components of a single inference process for offering confidence in the resulting projections and leading decision‐making in policy and investment. The climate modeling community provides a compelling example of how robust MIPs can enhance MMEs. Through projects like CMIP, poorly performing or biased models have been systematically identified with lower weights in ensembles and, in some cases, excluded from ensemble forecasts (Brunner et al. 2020; Sitch et al. 2024). This practice has improved the credibility and accuracy of climate projections and represents a best practice that the ecosystem modeling community should emulate.

Beyond their role in generating consensus projections, MMEs also serve a deeper purpose for the scientific community: to improve our understanding of system dynamics and model processes, identify errors in model implementation as well as gaps in scientific knowledge requiring further research (Boomer et al. 2013). This function is primarily achieved through MIPs, which enable iterative improvement of model representations and help identify data gaps in both observations and model inputs/constraints (Figure 1). Despite this potential, the contribution of MIPs in driving model improvement remains underutilized and underinvested in many ecosystem modeling communities (Maiorano et al. 2017; Sun, Fang, et al. 2021; Basso et al. 2025). There is a pressing need for attention to the broader scientific role of MIPs and MMEs beyond consensus projection.

BOX 1 **Definition of ecosystem models, MMEs and MIPs, and MIPs‐MMEs relationship.**

- Ecosystem models
  ○ Ecosystem models are representations of ecosystems that use mathematical formulations to describe, explain, or predict ecosystem dynamics by representing interactions among biotic (species, functional groups) and abiotic components (Geary et al. 2020), such as ecosystem responses to environmental and anthropogenic change. They range from simple conceptual frameworks to complex numerical simulations, with structure and complexity varying according to scientific and decision‐making objectives.
- Multi‐Model Ensembles (MMEs)
  ○ MMEs are approaches that combine predictions from multiple models with the expectation of improving robustness and quantifying predictive uncertainty.
- Model Intercomparison Projects (MIPs)
  ○ MIPs denote the coordinated scientific frameworks within which models are systematically compared, evaluated against observations, and analyzed to understand sources of agreement, divergence, and uncertainty.
- MIPs‐MMEs relationship:
  MIPs diagnose models against observations, whereas MMEs synthesize models for prediction; robust inference requires both.
  ○ Diagnostic function: MIPs primarily serve a diagnostic function, systematically evaluating model performance under standardized protocols and benchmarking against available observations. This diagnostic process benchmarks each model's strengths and weaknesses, quantifies uncertainties relative to known observations, and identifies structural or parametric deficiencies within individual models.
  ○ Prognostic prediction: MMEs are primarily prognostic tools designed to provide forward‐looking projections or to reconstruct unknown historical periods by aggregating outputs from multiple models, while also could also support diagnostic analyses of past variability and driver influences. The reliability of aggregated predictions from MMEs, for both future projections and historical reconstructions, depends directly on the rigor of the preceding MIPs.

In this perspective, we aim to identify current gaps and provide an objective and science‐based framework for the best practices of MMEs in ecosystem modeling, especially in the context of decision‐making. Ecosystem modeling is a broad category that includes many disciplines, including environmental science, plant/crop/forest science, soil science, biogeochemistry, biodiversity, and others. Some disciplines have done much in this space (especially those contributing to the IPCC reports, such as ecosystem carbon budget and fluxes; crop modelers have done extensive MIPs for crop yield through AgMIP), and some disciplines and topics have done more limited work. We will anchor our perspective based on the success and lessons learned from existing efforts by ecosystem modeling and climate/Earth system modeling communities (e.g., CMIP, AgMIP). This perspective focuses on four pillars: (1) theoretical foundations and challenges in ecosystem MMEs, (2) key considerations to implement rigorous MIPs‐MMEs, (3) MIPs as catalysts for model improvement and scientific advancement, and (4) actionable best practices guidelines, including adoption of standardized protocols for MIPs and MMEs, iterative model improvement and benchmarking through coordinated community collaboration, and co‐design of MIPs‐MMEs efforts with stakeholder engagement to align MMEs with scientific integrity and stakeholder relevance. Building on these efforts, this perspective presents a comprehensive conceptual framework for understanding uncertainties in ecosystem MMEs, highlights the issue of model inter‐dependence arising from shared legacy frameworks, critical roles of MIPs for guiding MME model selection/weighting and model improvement, and synthesizes these insights into best‐practice guidelines for designing and interpreting MIPs‐MMEs for decision‐making context.

## 2 Theoretical Foundations and Uncertainty Sources of MMEs

MMEs estimate targeted quantities by aggregating predictions from multiple models. Models are inherent simplifications of reality and, therefore, prone to error. When combined with uncertain forcing inputs and user‐dependent assumptions, these uncertainties cascade through the system to affect the final ensemble output. Mathematically, the errors can be decomposed into three independent parts (see Box 2 for detailed derivation):

(1) $$E_{\text{total}}=E_{\text{Bias}}+E_{\text{Covariance}}+E_{\text{Variance}}$$

where $E_{\text{total}}$ is the total error after ensembling, measured by the mean square error. $E_{\text{Bias}}$ is the bias, which quantifies the persistent systematic deviation of the ensemble mean from the true value, reflecting structural inaccuracies, calibration offsets inherent to the aggregated model simulations, and biased forcing. $E_{\text{Covariance}}$ represents the component of error arising from correlated uncertainties shared across models, such as common forcing datasets, similar structural assumptions, or shared theoretical frameworks. Because these errors are correlated, they cannot be reduced through simple averaging across models. $E_{\text{Variance}}$ represents the component of ensemble error arising from model‐specific, uncorrelated uncertainties, such as parameter choices, stochastic components, or internal variability that differ across models. These errors tend to cancel out as the ensemble size increases.

BOX 2 **Uncertainty decomposition in model ensembles.**

To rigorously evaluate the potential and limitations of MMEs, we decompose the ensemble error into its fundamental components. Using mean squared error (MSE) as the metric, the uncertainty of MMEs can be separated into two parts, as shown in Equation (1): (1) the bias‐related term, $E_{\text{Bias}}$, arising from the biases of individual models within the ensemble; and (2) variance‐related terms, $E_{\text{Covariance}}$ and $E_{\text{Variance}}$, originating from the variances of individual models and their mutual dependencies.

Model pairs within an ensemble are generally not independent. Independent and dependent noise components affect the final MME results differently, contributing to $E_{\text{Covariance}}$ and $E_{\text{Variance}}$, respectively. Model inter‐dependence can be quantified using correlation coefficients, which may vary across model pairs. In this box, we present the derivation of MME uncertainty under a simplified assumption that all model pairs share the same correlation. The derivation for the general case, allowing for pairwise‐varying correlations, is provided in Appendix A.

Let $f\left(x\right)$ denote the true function of the ecosystem, where x represents inputs such as precipitation or temperature. An ensemble consists of k models, where the prediction of the i‐th model for the m‐th sample is $\hat{f_{i}}\left(x_{m}\right)$. The error of any single model prediction, $\varepsilon_{i,m}$, is the deviation from the truth:

(2) $$\varepsilon_{i,m}={\hat{f}}_{i}\left(x_{m}\right)-f\left(x_{m}\right)$$

For individual models, the error can be decomposed into bias and variance terms,

(3) $$\varepsilon_{i,m}=\left(E_{f}\left[{\hat{f}}_{i}\left(x_{m}\right)\right]-f\left(x_{m}\right)\right)+\left({\hat{f}}_{i}\left(x_{m}\right)-E_{f}\left[{\hat{f}}_{i}\left(x_{m}\right)\right]\;\right)$$

Let

(4) $$\beta_{i,m}=E_{f}\left[{\hat{f}}_{i}\left(x_{m}\right)\right]-f\left(x_{m}\right),$$

(5) $$s_{i,m}={\hat{f}}_{i}\left(x_{m}\right)-E_{f}\left[{\hat{f}}_{i}\left(x_{m}\right)\right],$$

where $\beta_{i,m}$ represents the systematic bias (consistent over‐ or under‐estimation) of the ith model. The term $s_{i,m}$ denotes the model variance, and its mean and variance are 0 and $\sigma_{i}$.

The noise term can be further decomposed into two parts:

(6) $$s_{i,m}=\sigma_{i}\left[\sqrt{\rho }\cdot Z_{m}+\left(\sqrt{1-\rho }\right)\cdot \eta_{i,m}\right],$$

The first term of Equation (6) in the bracket represents the correlated variance (e.g., shared structural deficiencies or forcing data errors). ρ is the inter‐model correlation coefficient. $Z_{m}$ represents its randomness at the m‐th sample, and its mean and variance are 0 and 1, respectively. The second term of Equation (6) in the bracket represents the uncorrelated variance. $\eta_{i,m}$ represents its randomness for the i‐th model at the m‐th sample. Then, $\varepsilon_{i,j}$ is given by,

(7) $$\varepsilon_{i,m}=\beta_{i}+\sigma_{i}\left[\sqrt{\rho }\cdot Z_{m}+\left(\sqrt{1-\rho }\right)\cdot \eta_{i,m}\right].$$

The ensemble error for a single sample j, $\varepsilon_{\mathrm{ens},j}$, is the average of these individual errors,

(8) $$\varepsilon_{\mathrm{ens},m}=\frac{1}{I}\sum_{i=1}^{I}\left[\beta_{i}+\sigma_{i}\left(\sqrt{\rho }\cdot Z_{m}+\left(\sqrt{1-\rho }\right)\cdot \eta_{i,m}\right)\right].$$

The mean square error (MSE) is given by

(9) $$\mathrm{MS}E_{\mathrm{ens},k}=E_{m}\left[{\left(\varepsilon_{\mathrm{ens},m}\right)}^{2}\right]={\left(\frac{1}{I}\sum_{i=1}^{k}\beta_{i}\right)}^{2}+\rho {\left(\frac{1}{I}\sum_{i=1}^{I}\sigma_{i}\right)}^{2}+\frac{1-\rho }{I^{2}}\sum_{i=1}^{I}\sigma_{i}^{2}.$$

The equation then is rewritten as Equation (1), where

$$E_{\text{Bias}}={\left(\frac{1}{I}\sum_{i=1}^{I}\beta_{i}\right)}^{2},\;E_{\text{Covariance}}=\rho {\left(\frac{1}{I}\sum_{i=1}^{I}\sigma_{i}\right)}^{2},\;E_{\text{Variance}}=\frac{1-\rho }{I^{2}}\sum_{i=1}^{I}\sigma_{i}^{2}.$$

**FIGURE 2:**
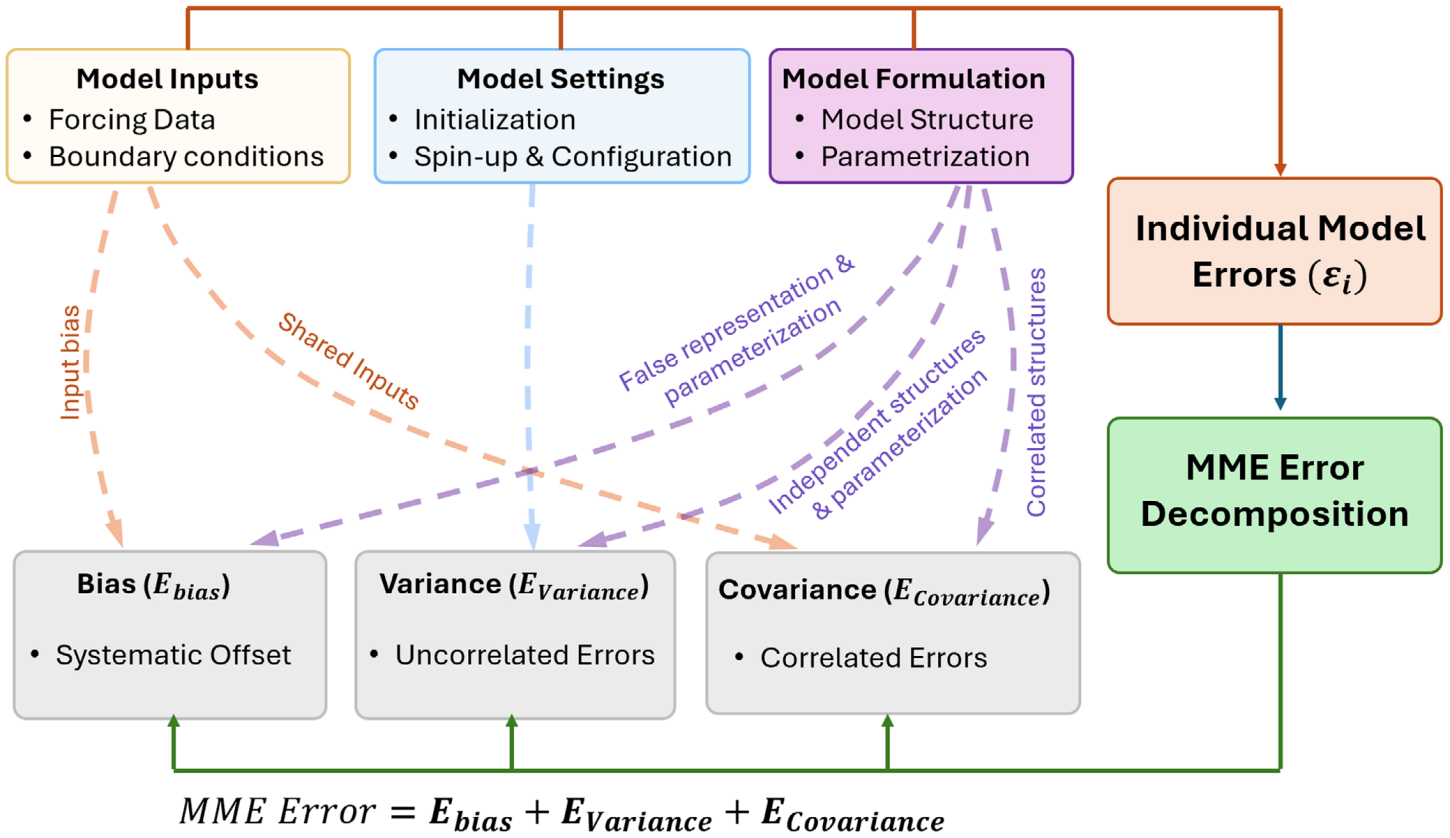
Conceptual Framework of Ecosystem Model Error, where MME refers to the Multi‐Model Ensemble, and the other terms are defined in the text.

Each term in Equation (1) is associated with one or more sources (Figure 2) in the ecosystem model ensemble, including (1) ensembles generated from a single model with different configurations (Deser et al. 2020; Morley et al. 2018), and (2) ensembles composed of multiple models (Bonan and Doney 2018; Dietze 2017). The uncertainty sources in ecosystem models can largely mirror those in Earth system models (ESMs), but also have their specific challenges. ESMs simulate all major interconnected components of the Earth's system, including the atmosphere, ocean, land, glacier, and biosphere (Bonan and Doney 2018); ecosystem modeling tends to focus on a subset of them, such as biosphere responses to the environment. Built up on existing work from ESM MMEs (Knutti and Sedláček 2013) and others (Wang et al. 2024), we summarize the following five principal uncertainty sources for ecosystem models:

1. *Internal variability (or initial condition uncertainty)* arises from the nonlinear behaviors of a complex system, where small (or even negligible) differences in initial states or boundary conditions can lead to large divergent outcomes (i.e., sometimes referred to as chaotic behavior of a system). This source of error can be treated as a form of model‐specific uncertainty and primarily contributes to $E_{\text{Variance}}$. It is typically addressed using ensembles of a single model with slightly perturbed initial conditions or inputs.
2. *Model input uncertainty* arises because critical model inputs carry uncertainty from imperfect measurements and retrieval algorithms, sparse or uneven observational coverage, temporal/spatial heterogeneity, and intrinsic stochasticity in the system (Bulgin et al. 2022). When a common input dataset is prescribed, this source of uncertainty is shared across all models. As a result, input errors tend to induce correlated errors among models, thereby contributing primarily to $E_{\text{Covariance}}$ and potentially also to $E_{\text{bias}}$ if the input dataset contains systematic errors. Because these errors are shared across models, they cannot be reduced through ensemble averaging. When input uncertainty is nontrivial, it should be explicitly propagated using multi‐input‐source‐ensemble approaches that integrate multiple data sources to capture a plausible range of input data.
3. *Model structural uncertainty* arises from incomplete scientific understanding and from alternative representations of real‐world processes in mathematical or algorithmic form during the model development process. This uncertainty might contribute to $E_{\text{bias}}$, especially when critical processes are omitted or misrepresented and errors cannot be corrected through calibration. The bias could be assessed in individual models by statistically evaluating biases against observations (Ogle et al. 2007). MMEs can further help mitigate this uncertainty by leveraging models that emphasize different processes or adopt different approximations. However, their effectiveness may be limited when models are interdependent and share similar or identical structures, for example, when developers rely on common theoretical frameworks or reference implementations. Consequently, model structure uncertainty can contribute to $E_{\text{bias}}$, $E_{\text{Covariance}}$ (through dependent or correlated structures) and $E_{\text{Variance}}$ (through independent structural differences).
4. *Model parameter uncertainty* pertains to the selection and accuracy of parameter values within models. Model parameter uncertainty arises from variability and limited knowledge of both process‐specific and location‐specific parameters (Guan et al. 2023). This source of error can be treated as a form of model‐specific uncertainty and primarily contributes to the $E_{\text{Variance}}$ and $E_{\text{bias}}$. $E_{\text{Variance}}$ is usually examined by perturbed parameter ensembles, sets of single‐model simulations with different choices for various parameters. To mitigate these errors, model‐data fusion techniques are often employed, which directly reduce $E_{\text{bias}}$ through systematic adjustment and constrain the parameter space to minimize $E_{\text{Variance}}$ (Dietze et al. 2018; Guan et al. 2023; Gurung et al. 2020; Luo et al. 2015; Peng et al. 2011).
5. *Scenario uncertainty* arises from the wide range of possible future conditions under which ecosystems may operate, including changes in climate, land use, policy, market forces, and management practices (Reilly and Willenbockel 2010). These uncertainties are external forcings to the models themselves but significantly shape the projections they generate. This source of uncertainty is shared across all models and therefore primarily contributes to the $E_{\text{Covariance}}$.

## 3 Key Considerations of MMEs for Ecosystem Modeling

Based on the theoretical framework of MMEs and the five major uncertainty types outlined in Section 2, this section will discuss key considerations for conducting decision‐making relevant MMEs of ecosystem modeling.

### 3.1 Decision‐Making‐Relevant MMEs Require Intentional Design: MMEs Quality Depends on MIP Rigor, Especially When There Is Limited Availability of Models

**FIGURE 3:**
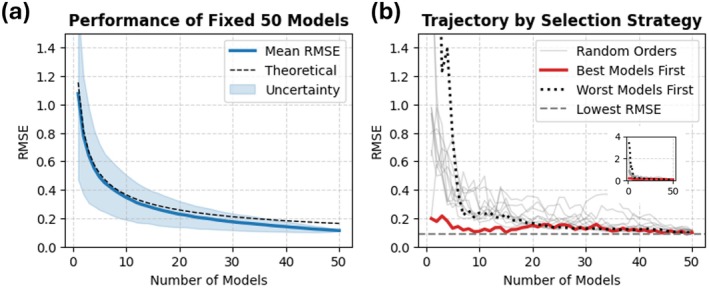
Effectiveness of MMEs and the impacts of model selection, based on the theoretical framework in Box 2 with detailed experimental settings and derivations in Appendix B. (a) The change in performance of MME with increasing ensemble size. The solid blue curve shows the mean RMSE computed over 1000 trajectory samples drawn from 50 candidate models as the ensemble size increases. The shaded region denotes ±1 standard deviation across samples. The dashed line indicates the theoretical expectation. Overall, with the increase in ensemble size, the mean RMSE decreases. The shaded area indicates substantial variability, particularly for small ensemble sizes. (b) Ten individual trajectories of the RMSE as models are progressively added to the ensemble. The red line represents adding models from the best (lowest RMSE) to the worst (highest RMSE). The dashed black line represents adding models from worst to best. The other eight trajectories are selected randomly for illustration. The red line indicates the lowest ensemble RMSE for adding models from the pool of best performing models. Overall this simulation shows that the performance of MMEs could benefit from prior information from MIPs, and intentionally choosing better‐performing models leads to improved MME results, especially with a low number of models.

“How many models are enough?” is a persistent question in MMEs (Asseng et al. 2013). Using the theoretical framework from Section 2, we show that, on average, MME errors statistically decrease with an increasing number of models (Figure 3a). This statistical finding is generic and does not depend on a specific application domain, which confirms various reported MME studies across ESM/climate and crop yield modeling (Hagedorn et al. 2005; Huntzinger et al. 2013; Knutti et al. 2010; Martre et al. 2015; Schwalm et al. 2015). It provides a theoretical basis for the common assumption that more models tend to improve MME performance. In climate science, the CMIP routinely involves 20 or more models for different sub‐MIPs, enhancing diversity through variance cancellation (Eyring et al. 2016). In ecosystem modeling, however, such large ensembles are rarely feasible due to limited model availability. Thus, decision‐making‐relevant MMEs aiming to achieve the best performance under the practical constraints (e.g., availability of models) should pay more attention to model choice, ordering, and composition.

Our theoretical framework further shows that choosing models ranked based on better‐to‐worse individual performance (through MIPs) leads to improved MME performance (Figure 3b), a pattern that is particularly pronounced when only a limited number of models are involved (left side in Figure 3b). Further revealed by Figure 3b, we find that with such performance‐informed model selection, increasing the number of models does not necessarily reduce MME error. In contrast, including poorly performing models can degrade overall MME performance. These results collectively highlight the importance of MIPs to provide a standard evaluation of individual model performance against a wide range of benchmark observations and environmental conditions. MIPs are thus essential for informing model selection and weighting, enabling MMEs to achieve decision‐making‐relevant goals to get better MME results under practical constraints (especially with limited availability of models).

MIPs also provide a mechanism to encourage innovation and allow better models to stand out (Eyring et al. 2016; Huntzinger et al. 2013; Wang et al. 2017). In some cases, MIPs might identify a single high‐performing model that may even outperform all the rest. A notable example comes from the computer vision and machine learning area: for the ImageNet annual competition in 2012, a convolutional neural network, AlexNet, achieved a shockingly better performance on the benchmark data set of more than 7.3 percentage points lower in a top‐5 error than the rest of the best models at that time (Krizhevsky et al. 2017). This event finally shifted Artificial Intelligence from traditional feature engineering toward the era of deep learning. Thus, rigorously designed MIPs (e.g., ImageNet) can enable innovation rather than averaging or muting innovation.

It is worth noting that our argument does not advocate single model dominance in general. In most applications, inclusion of more models generally leads to better MMEs (Figure 3a). Rather, we emphasize that rigorously designed MIPs are essential to fairly evaluate individual models and provide information to support the construction of decision‐making‐relevant MMEs that reflect decision context, hypothesis diversity, and scale‐specific needs (den Boon et al. 2019; Clark et al. 2011; Rudin 2019), especially when only a limited number of models are involved. Furthermore, we caution that MIP rankings of models may change under different environments or conditions (Asseng et al. 2013; Piao et al. 2013), thus a robust MIP should be done to include a wide range of environmental conditions that MME scenarios need to cover.

### 3.2 Redundancy and Similarity Among Models Reduce MME Effectiveness

**FIGURE 4:**
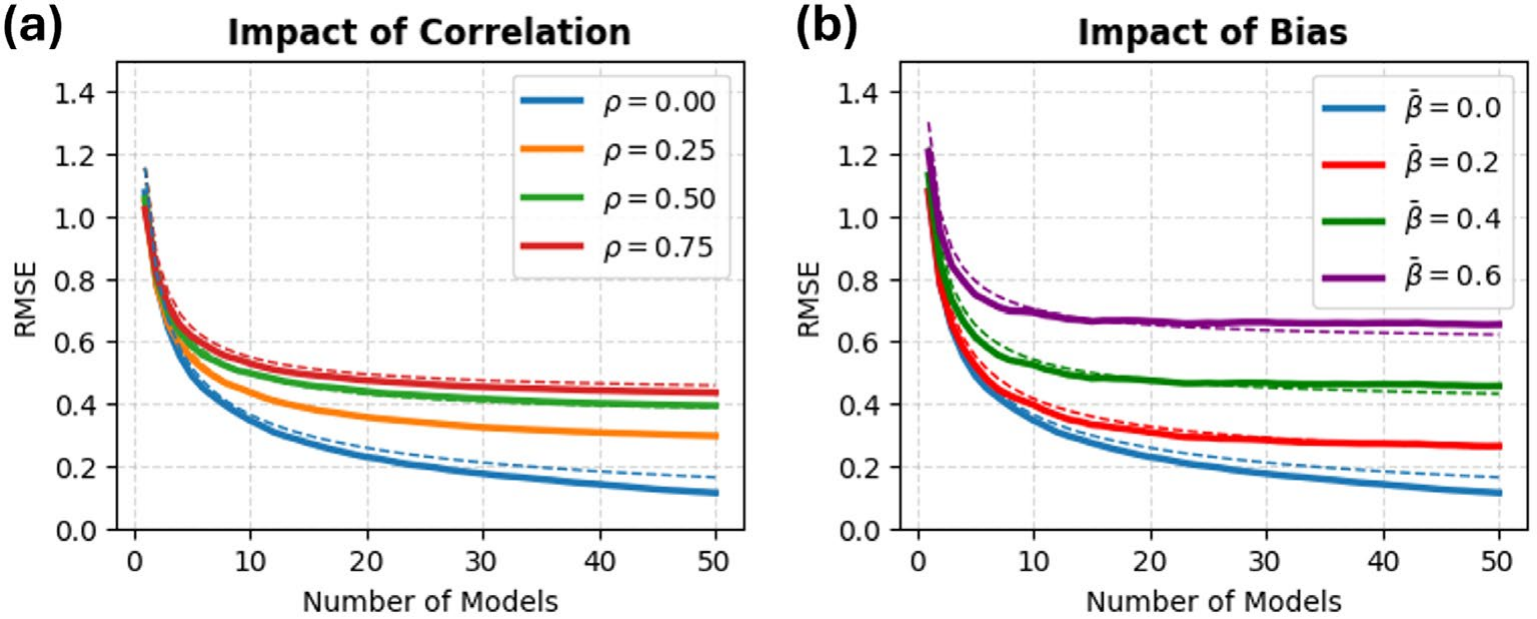
The impact of model inter‐dependence and systematic bias on the effectiveness of MMEs, based on the theoretical framework in Box 2 with detailed experimental settings and derivations in Appendix B. The solid curves show the mean RMSE computed over 1000 trajectory samples drawn from 50 candidate models as ensemble size increases. The dashed line represents the theoretical expectation. (a) RMSE decreases as the ensemble size increases, but the rate and magnitude of this reduction depend on model inter‐dependence. Larger ρ indicates stronger model inter‐dependence and lower effective independence among models. When *ρ* = 0, model errors are independent and RMSE declines rapidly with increasing ensemble size. As *ρ* increases from 0.25 to 0.75, the decline in RMSE becomes progressively weaker, and the RMSE remains higher even when many models are included in the ensemble. (b) RMSE decreases as the number of models in the MME increases, but remains higher when the average systematic bias is larger. Here, $\bar{\beta }$ represents the average systematic bias across models, defined as the mean tendency of the candidate models to consistently overestimate or underestimate the target variable. As $\bar{\beta }$ increases from 0.2 to 0.6, the RMSE curves shift upward, indicating that the bias‐induced model uncertainty is not reduced by MMEs.

Correlated models (due to structural similarity, same original roots, etc.) would significantly compromise the performance benefit of MMEs, as further inferred from our theoretical framework (Figure 4a). Our finding shows that MME performance decreases with the increase of model dependence (Figure 4a). This finding provides strong evidence that MMEs' performance is also influenced by the independence of the available models and should be interpreted accordingly.

**FIGURE 5:**
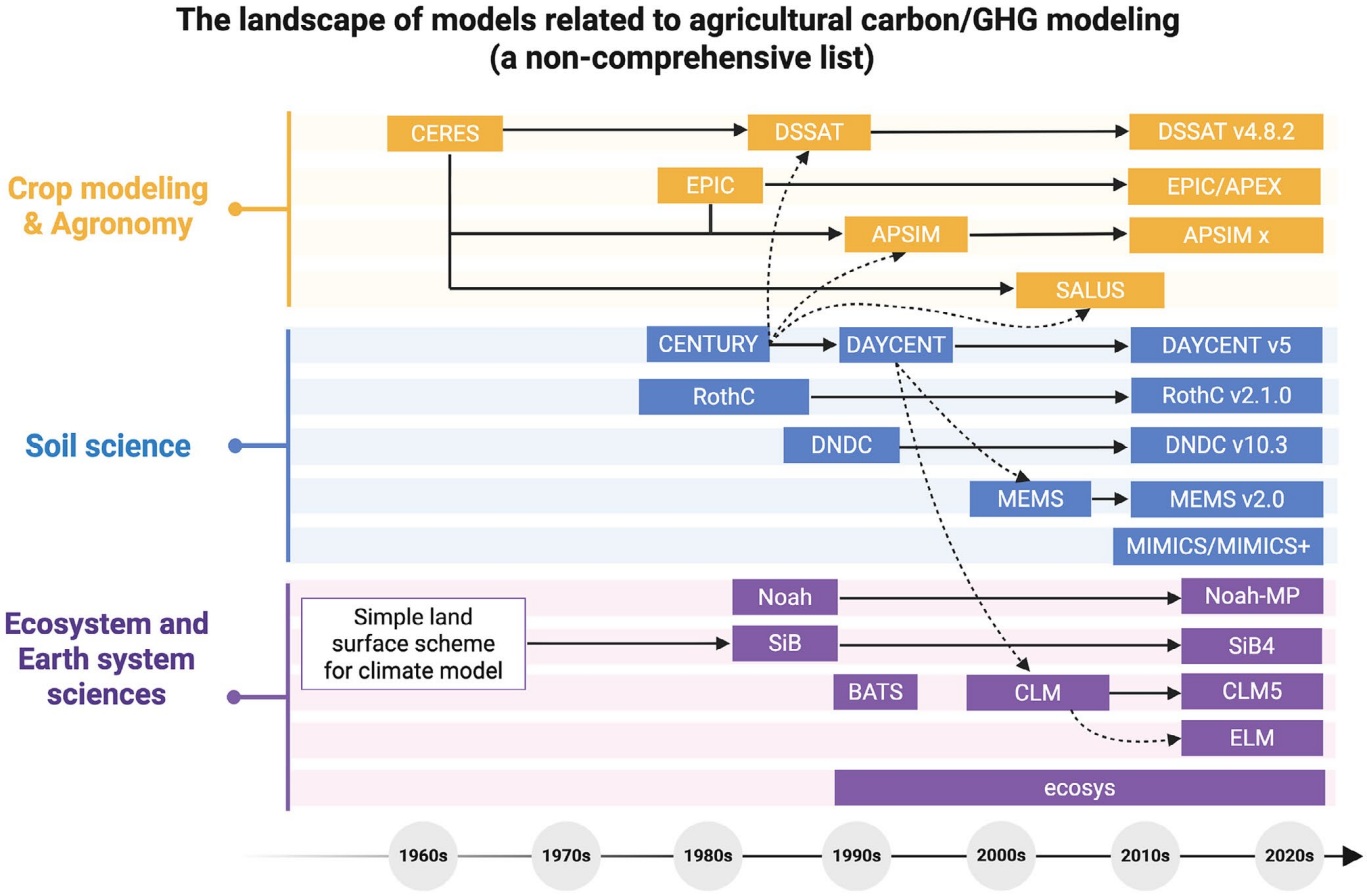
Examples of different models related to agricultural carbon/GHG modeling and their evolutions, developed by different scientific communities. This is a non‐comprehensive list, but highlights the legacy of some models sharing similar original roots. Solid lines denote version succession within a model, whereas dashed lines indicate inheritance and/or reference relationships between different models.

Many ecosystem models share common roots in legacy frameworks (Figure 5), such as soil biogeochemical processes from CENTURY (Parton et al. 1988), or crop growth processes from CERES (Ritchie and Otter 1985), thus compromising model inter‐independence and potentially constraining the effectiveness of MMEs (Figure 4a). Some of these legacy frameworks were developed in the 1970s and 80s, and their core structures remain largely unchanged and continue to be adopted in many other models. Such redundancy of shared model structures in existing MIPs may lead to systematic biases and correlated errors. Aggregating structurally similar models (or overweighting one hypothesis class) without scrutiny of the model inter‐dependence risks creating the apparent perception of robustness but in reality inflating the MME errors (den Boon et al. 2019). Beyond shared legacy frameworks, model inter‐dependence may also arise from the use of common forcing datasets, calibration data, or shared theoretical assumptions across models, further increasing the likelihood of correlated errors.

### 3.3 Parametric and Structural Uncertainties Are the Major Sources of Uncertainties for Ecosystem Models

Model parameters and model structure remain as the major sources of uncertainties for ecosystem models (Bonan et al. 2019; Shi et al. 2018; Zaehle et al. 2005; Wang et al. 2024), and both can introduce systematic biases in model results. Our theoretical framework shows that including models with large biases in the MIPs would limit MMEs performance (Figure 4b). Specifically, large individual model biases propagate into the ensemble, resulting in poor MMEs performance. This finding underscores the importance of MIPs that evaluate and quantify the model biases with benchmark observations, while also highlighting the necessity of correcting model bias before MME ensemble construction as well as quantifying any residual bias and propagating to MME results. Reducing model biases requires reducing uncertainty in both model parameters and model structures.

To reduce uncertainties from model parameters, conducting necessary model calibrations can effectively reduce systematic bias before getting into MMEs. It is worth noting that model calibration in the MIPs‐MMEs context should be clearly differentiated from the unrecommended practice of “tuning model parameters simply to get a better fit of model simulation with observations”. The key difference here lies in the following aspects, which is also reflected in the general trend of modernizing process‐based modeling (Tang et al. 2024) that advocates: (i) inclusion of sufficient complexity of model mechanisms for processes (Challinor et al. 2014), (ii) use of physically‐meaningful parameters and model representation of fluxes and pools (which ideally can be directly measured), (iii) focus on capturing internal causal responses of modeling variables to environmental responses (e.g., photosynthesis responses to elevated CO_2_) or internal interactions among different variables (e.g., N_2_O emission to soil oxygen content), rather than only aiming for a better alignment to the pure value of some observations (e.g., a better fit with crop yield); and (iv) differentiation of two types of parameters: location‐specific parameters to reflect site unique dependence like soil properties and cultivar growth, and process‐specific parameters to represent general system behaviors that can transfer across different environments (Guan et al. 2023). Maintaining the above distinction for model calibration is also essential for transparency and reproducibility, particularly in policy‐facing applications where the credibility of model‐based assessments depends on clearly documented calibration procedures.

A persistent challenge to quantifying and reducing model parameter uncertainty is equifinality, whereby different parameter combinations (and sometimes compensating structural errors) can reproduce the same observations (Beven 2006). The ecosystem modeling community could borrow the perturbed parameter ensembles methodology in climate modeling. Constraining this uncertainty requires information‐rich observations and distinct calibration strategies: site‐level observations for location‐specific parameters, and meta‐analyses or experimental studies for process‐specific ones. Model‐data fusion techniques such as Bayesian calibration (Keenan et al. 2011; Raupach et al. 2005; Gurung et al. 2020) and emerging AI‐driven methods (see Section 3.6) offer promising avenues to reduce these uncertainties.

Model structural uncertainty in ecosystem modeling is also a key challenge (Bonan and Doney 2018; Wang et al. 2024). Ecosystem processes, such as soil organic matter decomposition, nutrient cycling, hydrological flows, or species distribution and interactions, all have some long histories of model development, creating much legacy but also potential inertia for innovation in many cases. For example, to model soil moisture limitation on ecosystem productivity, the Jarvis‐type of empirical water stress functions (Jarvis 1976) has a ~50 years history and is still the dominantly used parameterization with static coefficients in many ecosystem/crop models (Combe et al. 2016; Sloan et al. 2021; Yang, Guan, et al. 2025); while some recent models adopt trait‐based plant hydraulic schemes (Christoffersen et al. 2016; Eller et al. 2018; Xu et al. 2016; Yang, Guan, et al. 2025), representing more first‐principle processes. Soil carbon cycling was modeled using linear turnover‐based depictions by most models since the 1970s (Jenkinson and Rayner 1977; Parton et al. 1988), but more recently, microbe‐explicit formulations have been developed and shown their advantages (Chandel et al. 2023; Koven et al. 2013; Sihi et al. 2016; Sulman et al. 2018). Incomplete (or missing) representation of essential process knowledge in existing models further complicates model predictions. Processes like microbial mechanisms or soil mineral‐organic matter interactions, or interactions among multiple environmental stressors that carry critical causal relationships between models' state variables are often oversimplified or absent (Tang et al. 2024). This insufficiency limits the models' ability to capture critical dynamics, especially for context‐sensitive processes like nitrous oxide (N_2_O) emissions (Tian et al. 2018) or long‐term soil organic carbon (SOC) change (Smith et al. 1997). In many cases, innovation of ecosystem models is overdue, modification or addition is urgently needed, and sometimes an overall revamp to develop a new structure and framework of ecosystem models to catch up with other Earth system model components is also justified.

### 3.4 Critical Role of Benchmark Data in MIPs‐MMEs and General Data Scarcity Challenges

Robust MIPs‐MMEs depend on high‐quality, standardized benchmark datasets. In MIPs, benchmarking is used to evaluate and improve individual models against observations, reducing model‐specific errors prior to ensemble construction; the extent to which parameter uncertainty can be reduced depends on the information content of observational constraints (Keenan et al. 2013). In MMEs, benchmarking is used to assess ensemble‐level performance and residual uncertainty after aggregation, requiring observations with sufficient spatiotemporal coverage to evaluate final outcomes. Models that are poorly calibrated or lack validation against observational data introduce errors that ensemble averaging cannot resolve.

In ecosystem research, important progress has been made through coordinated observational networks such as FLUXNET/AmeriFlux (Baldocchi et al. 2001, 2024), NEON (Schimel et al. 2007), LTER (Hobbie et al. 2003), and LTAR (Walbridge and Shafer 2011), which provide long‐term observations of carbon, water, and energy fluxes across diverse ecosystems, thereby underpinning benchmark datasets in ecosystem research. FLUXNET/AmeriFlux and NEON further provide harmonized flux measurements through standardized instrumentation, data‐processing pipelines, and quality‐control protocols, enabling consistent cross‐site and cross‐biome comparisons. For example, FLUXNET/AmeriFlux eddy‐covariance observations have enabled global syntheses of terrestrial carbon cycling and ecosystem–atmosphere exchange (Beer et al. 2010; Jung et al. 2011; Luo et al. 2025). The LTAR network also has supported transdisciplinary analyses linking management practices with agroecosystem sustainability (Tsegaye et al. 2024). Voluntary community data collectives like the TRY Database (Kattge et al. 2011), SAPFLUXNET (Poyatos et al. 2016), International Soil Carbon Network (ISCN) (Nave et al. 2016), International Soil Radiocarbon Database (ISRaD) (Lawrence et al. 2020), Continuous Soil Respiration (COSORE) Database (Bond‐Lamberty et al. 2020), and open‐sourced database platforms (such as https://odjar.org/) also provide useful sources to store, archive, and share benchmark data for ecosystem modeling.

However, benchmark data remain incomplete for many ecosystem studies, limiting systematic evaluation and improvement of model performance across sites and systems (Lovenduski and Bonan 2017; Ruane et al. 2017). Bridging this divide requires stronger coordination between modelers and experimentalists to align data collection with modeling needs (Schuwirth et al. 2019; Wang et al. 2024). A system‐level approach is essential to account for both fluxes (e.g., greenhouse gas emissions, nutrient leaching) and pools (e.g., soil carbon, biomass) to fully capture ecosystem dynamics (Guan et al. 2023). For example, SOC modeling suffers from a lack of long‐term paired experiments and delta SOC measurements, which are critical for detecting meaningful changes over time (González‐Sosa et al. 2024; Maas and Sihi 2025; Ogle et al. 2023; Sainju et al. 2024; West and Post 2002). Similarly, modeling N_2_O emissions demands high‐resolution, spatially and temporally explicit data, as infrequent measurements (e.g., biweekly) can miss significant emission events and lead to large uncertainties (Del Grosso et al. 2022). Addressing these data gaps is key to enabling more accurate and policy‐relevant ecosystem modeling.

Benchmark datasets are also not error‐free: observations embed measurement error (instrument/observer limitations) and can also reflect systematic error from biased sampling or protocols, especially when key processes are incompletely understood (Regan et al. 2002). In complex, heterogeneous systems, important relationships can vary across space and time, and some quantities are only indirectly observed via surrogates, so benchmarking can unintentionally foster “spurious certainty” if these limitations are ignored (Biggs et al. 2009). A multi‐model (competing‐hypotheses) framework can add value beyond evaluation by helping prioritize what, where, and when to measure; that is, informing targeted monitoring that focuses effort on observations most informative for discriminating among alternative model explanations and reducing decision‐relevant uncertainty (Nichols and Williams 2006).

### 3.5 Other Considerations in Uncertainty Mitigation and Implementation of MMEs in Ecosystem Modeling

Initial conditions, while being a major source of uncertainty in climate and weather models due to the chaotic nature of atmospheric dynamics (Baker and Gollub 1996), tend to play a comparatively smaller role in ecosystem models. This difference reflects the generally slower temporal dynamics, less internal feedbacks and nonlinearities of ecological processes compared to climate or weather systems (Deser et al. 2020). As a result, initial conditions are often of lesser concern for ecosystem modeling, especially for annual cropping systems. However, in ecosystems dominated by long‐lived organisms (e.g., forests) and in applications where long‐term soil carbon dynamics are a primary focus, inaccurate initialization can lead to drift and biased projections, making careful treatment of initial conditions essential for specific processes, timescales, and management contexts.

Input uncertainty can be significant for ecosystem modeling, particularly when assessing location‐specific impacts, although these errors are typically smaller than uncertainties associated with model parameters and structure (Ogle et al. 2010). Regardless, they should not be ignored in MMEs. For example, agroecosystem modeling heavily relies on a range of input data, including environmental factors, management practices, and crop characteristics (Guan et al. 2023). Although large‐scale soil and weather databases are available, their spatial resolution, coverage, and accuracy vary significantly across regions and sources (Hersbach et al. 2020; Tifafi et al. 2018; Zhou et al. 2023). Meanwhile, management data are highly variable across space and time, and are often not systematically recorded or readily accessible, posing a major source of input uncertainty for ecosystem models (Guan et al. 2023; Tubiello et al. 2013). Consequently, for applications that are highly sensitive to input data, particularly in data‐limited regions, MMEs may benefit from explicitly evaluating uncertainty across various sources of data inputs.

Unlike structural or input uncertainties that largely pertain to modeling scientists only, scenario uncertainty reflects plausible but unpredictable trajectories, making it essential to engage stakeholders (such as farmers, policymakers, and practitioners) in the scenario development process (Lambin et al. 2000). Co‐developing scenarios with both modeling scientists and stakeholders ensures that they are not only scientifically rigorous and feasible but also relevant and acceptable to end users (McMahon et al. 2015). This process requires mutual collaboration: scientists must translate complex system dynamics into transparent and credible storylines, while stakeholders contribute contextual knowledge, preferences, and tolerance for uncertainty (Coppess et al. 2025). Effective collaboration helps ensure that modeled scenarios are both realistic and actionable, enhancing their value for planning, adaptation, and decision‐making under uncertain futures.

### 3.6 Enhancing MMEs Through Artificial Intelligence and Machine Learning (AI/ML)

With the acceleration of AI/ML engagement in all aspects of scientific research and practical applications, we discuss how AI/ML could support MIPs‐MMEs to address specific technical challenges related to uncertainty quantification and reduction, computational constraints, and workflow management. We list the following five aspects that could improve MME performance. While highly promising (Jin et al. 2026), we acknowledge that AI/ML approaches still need to overcome challenges of model interpretability, substantial data requirements for training and validation, and governance considerations such as transparency and reproducibility.

1. Surrogate modeling for Ensemble expansion. AI/ML emulators (or surrogates) function as computationally efficient alternatives to full physical models, which are useful for meeting the requirement for large ensemble sizes in MMEs. Rather than repeatedly running the full physical model, AI/ML emulators trained on physical model‐generated simulations can efficiently approximate model outputs. This step bypasses the computational bottleneck, enabling the generation of massive ensembles to capture rare tail events and explore parameter uncertainty efficiently (Tebaldi et al. 2025; Yang, Elsaesser, et al. 2025).
2. Reduction of systematic model bias. AI/ML strategies, such as ML‐based bias correction, residual learning, transfer learning, could be utilized to enhance MMEs by mitigating model bias within each ensemble, resulting from necessary approximations and under‐calibration. First, AI/ML emulators grounded in physical model outputs enable fine‐tuning on observational data to reduce bias, which can align their predictions closer to reality than the physical model, especially by reducing structural uncertainty. To be noticed, the fine‐tuning should only be conducted over high quality data that represents the general processes, and should use preserved or regularized learning methods to retain prior knowledge and prevent overfitting (He et al. 2024; Liu et al. 2024). Further, ML components can be used to replace specific error‐prone parametrizations (e.g., cloud microphysics and soil moisture) within the physical model. By learning directly from high‐fidelity data, these components correct biases at the source process level while maintaining the model's overall physical consistency (Hess et al. 2023; Kochkov et al. 2024; Zhang et al. 2024).
3. Dynamic model weighting. AI/ML classifiers can create a “smarter” ensemble that surpasses the performance of individual models and a simple average. Specifically, AI could dynamically assign weights based on spatial–temporal performance analysis from MIPs, which accounts for the fact that model accuracy may fluctuate across different regions and timeframes (Bai et al. 2021; Chen et al. 2023; Sharma et al. 2026; Sun, Scanlon, et al. 2021). Instead of using static weights, AI identifies complementary strengths to prioritize the most reliable models for specific conditions, which mimics the MIP processes. Ultimately, advanced AI analysis uncovers the unique regimes where specific models provide the most informative predictions, maximizing the collective skill of the ensemble.
4. Probabilistic uncertainty quantification. Traditional MMEs often underestimate risk by using ensemble spread as a proxy for uncertainty, failing to propagate the internal errors of individual models. AI post‐processing, such as Bayesian Neural Networks or Quantile Regression Forests, can rectify this conflation by transforming raw outputs into full probability distributions (Hajihashemi and Shen 2024; Zhou et al. 2025). This approach explicitly partitions aleatoric uncertainty (stochastic noise) from epistemic uncertainty (model ignorance), yielding better‐calibrated confidence intervals. Furthermore, employing AI as a differentiable surrogate allows for the backpropagation of uncertainty, tracing predictive errors directly back to specific individual models or input drivers to identify the root causes of ensemble instability.
5. Automated workflow configuration. The complexity of MMEs often stems from manual, repetitive tasks, including data retrieval, configuring varied model parameters, and managing execution on high‐performance computing clusters. Large Language Model (LLM)‐driven AI agents hold the potential to address this challenge by autonomously planning and executing the entire workflow. They can proficiently handle data retrieval and the intricate configuration of multiple models, thereby automating the MME setup. This automation significantly minimizes human error and substantially speeds up the launch of large‐scale experiments (Ma et al. 2025; Xin et al. 2025).

## 4 MIPs‐MMEs as Catalysts for Model Improvement and Scientific Advancement

For scientists, properly designed MIPs offer the most effective mechanism for deepening our understanding of ecosystem processes and refining the models that simulate them. However, despite this potential, the role of MIPs in model development remains underutilized and underinvested. While many MIPs initiatives succeed in comparing model results and aggregating them, they often fall short in addressing the “so what” questions that push the field forward. Too frequently, MIPs conclude with comparative assessments without translating those insights into actionable steps for improving the models themselves.

Effective MIPs should go beyond comparison; they identify data gaps, missing or deficient process representations, and structural limitations in existing models (Eyring et al. 2016; Hall et al. 2019; Sherwood et al. 2020; Tokarska et al. 2020; Wang et al. 2017). These diagnostic insights can directly inform new data collection efforts and experimental designs, targeting areas where observational constraints are weak or where model uncertainties are highest. This insight creates a feedback loop: improved data collection and process understanding inform better modeling processes, and improved models, in turn, reveal where new data or process representation are most needed. This iterative process strengthens not only the scientific foundation but also the predictability of ecosystem models over time. It is worth noting that the Department of Energy Environmental System Science program has championed an Integrated Model‐Observation‐Experiment (ModEx) approach (ModEx Approach 2021) to achieve similar goals, though such a coordinated effort is rare beyond that community. Moreover, MIPs provide a standardized framework for continuously evaluating new model developments, allowing the modeling community to quantify improvements transparently and consistently. This ongoing evaluation ensures that progress in model refinement is not anecdotal or isolated but systematically assessed across studies and model generations.

Importantly, MIPs can yield outcomes that extend beyond ensemble construction or individual model refinement. They can lay the groundwork for enhanced modeling infrastructure through two complementary pathways. First, they can catalyze the development of community‐level models that integrate the strengths of multiple frameworks into a single, robust platform. The Community Earth System Model (CESM), developed through broad collaborations among various communities and hosted at the National Center for Atmospheric Research (NCAR), exemplifies this approach in the ESM domain (Danabasoglu et al. 2020). CESM integrates components from multiple research groups worldwide, ensuring that the best available science is incorporated and continuously updated within a shared infrastructure. Second, MIPs can lead to community‐supported infrastructure platforms that provide standardized frameworks, protocols, and tools to enable broad participation of different modeling groups to join MIPs‐MMEs activities without requiring model consolidation. Such digital infrastructures, including confidential clean room computing environments, can host heterogeneous models within a common evaluation framework, enabling systematic intercomparison without requiring users to directly run each model while preserving model ownership and intellectual property (Shuai et al. 2025). These platforms can lower barriers to MIPs‐MMEs participation while preserving modeling diversity and institutional independence. Adopting either or both strategies in ecosystem modeling, whether through shared open‐source models or interoperable infrastructure platforms, could substantially enhance model quality, transparency, and credibility. Such approaches would not only complement specific MIPs‐MMEs efforts, but also foster collaboration and innovation across research groups with varying institutional contexts and scientific priorities. Furthermore, both pathways would enable the identification of emergent relationships between observable metrics and uncertain future responses, using traceable benchmarking frameworks to guide model development toward more policy‐relevant outcomes (Hall et al. 2019; Sherwood et al. 2020; Tokarska et al. 2020).

Overall, MIPs and MMEs can be understood within the Expectation–Maximization paradigm (Dempster et al. 1977), in which MIPs diagnose model performance, inter‐dependence, and bias to establish the most robust modeling framework that is feasible, and MMEs integrate this information to produce predictions and uncertainty estimates. Iterating between these roles establishes a self‐improving modeling cycle, emphasizing that MME credibility depends on rigorous evaluation and feedback rather than ensemble size alone.

## 5 Best Practices of MMEs in Ecosystem Modeling

### 5.1 Best Practice 1: Robust MMEs Must Be Built on Rigorous MIPs

The first and most critical principle for constructing reliable MMEs is to anchor them on robustly executed MIPs (Eyring et al. 2016; Knutti et al. 2010; Rosenzweig et al. 2013; Schwalm et al. 2015). Without a solid diagnostic foundation, ensemble aggregation lacks the necessary context to ensure meaningful or trustworthy results. A well‐designed MIP provides the essential evaluation framework to assess model performance, diversity, and credibility before any aggregation takes place.

To achieve this, standardized protocols are needed for both MIPs and MMEs (Box 3). These protocols should define consistent procedures for initializing, running, calibrating, comparing, and evaluating models (Collier et al. 2018; Eyring et al. 2016; Rosenzweig et al. 2013). Standardization ensures that comparisons across models are valid and reproducible, allowing for systematic quantification of each model's strengths and weaknesses, benchmarked against historical data or observations.

High‐quality benchmark datasets are indispensable for this process (Beadling et al. 2026; Collier et al. 2018). These datasets ideally should span the spatial and temporal gradients representative of the systems being modeled and should include key state and flux variables that reflect the ecological processes under study. Networks, voluntary community data, as well as open‐sourced database platforms offer critical observational databases for ecosystem modeling, providing the data needed to validate model outputs against reality and assess model fidelity. Rigorous integration of satellite and airborne remote sensing observations with these in situ observations is also essential to augment data availability and constrain model uncertainty across spatial scales (Asner and Martin 2016; Guan et al. 2023; Schimel et al. 2015; Wang, Guan, Zhang, Jiang, et al. 2023; Wang, Guan, Zhang, Zhou, et al. 2023).

We will learn from the success of MIPs‐MMEs efforts. AgMIP has demonstrated the value of harmonized protocols, common datasets, and coordinated multi‐model experiments for model evaluation and uncertainty assessment (Rosenzweig et al. 2013; Wang et al. 2024). Likewise, a history of climate model evaluation (Eyring et al. 2019; Hassler et al. 2026; Randerson et al. 2009) has demonstrated the utility of systematic assessment for quantifying and reducing uncertainties in models. Community efforts in formulating internationally accepted model benchmarks (Hoffman et al. 2017), coordinate model intercomparison initiatives such as AgMIP’s Global Gridded Crop Model Intercomparison (GGCMI), and developing evaluation software frameworks, including the International Land Model Benchmarking (ILAMB) package (Collier et al. 2018) and platforms like modelevaluation.org and the CMIP Rapid Evaluation Framework (Hoffman et al. 2025), further illustrate how diverse benchmark datasets can be systematically curated (e.g., Obs4MIPs, Waliser et al. 2020) and applied to rigorous multi‐model evaluation and benchmarking. For example, ILAMB and the corresponding International Ocean Model Benchmarking (IOMB) package (Fu et al. 2022) were used to evaluate and intercompare historical simulations from two generations of land and ocean models (CMIP5 versus CMIP6), revealing improvements in the relative performance of newer models across a wide range of observationally constrained reference data (IPCC 2023, chapter 5, figure 5.7).

Comprehensive model benchmarking should not be limited to mean performance metrics but should also evaluate diurnal, seasonal, and interannual variability; variable‐to‐variable or functional relationships (Collier et al. 2018); process fidelity; responses to perturbation based on manipulation experiments (Wieder et al. 2019); and emergent system properties, capturing the complex dynamics of ecological systems (Abramowitz 2012; Luo et al. 2012), particularly those that are directly relevant to stakeholders' needs (e.g., soil, plant, and microbial responses to extreme events).

The outcomes of rigorous MIPs should directly inform the design of MMEs. Specifically, MIP results should guide: (1) *model selection*: determining which models to include or exclude based on their performance; (2) *model weighting*: defining how to appropriately weigh different models in the ensemble, recognizing that not all models contribute equally to reducing uncertainty. Simple averaging treats all models equally (Knutti et al. 2017; Merrifield et al. 2020), which may ignore potential differences in their performance or credibility. More sophisticated approaches, such as skill‐based Bayesian model averaging (Duan et al. 2007) or dynamic model weighting (see Section 3.6), can account for model reliability and uncertainty, leading to potentially more robust ensemble predictions.

BOX 3 **Major considerations of MIP and MME protocols.**

- High‐quality benchmark data:
  ○ Invest in long‐term databases for measurements reflecting key ecological processes of carbon, water, energy and nutrient cycles to facilitate MIPs‐MMEs, as well as for high‐quality forcing/input data (e.g. fine‐scale climate, soil data).
  ○ Require explicit reporting of data uncertainty, including measurement error, sampling bias, spatiotemporal representativeness, and known data gaps, to enable transparent model evaluation and uncertainty propagation.
  ○ Promote standardization of protocols for field measurements, metadata reporting, and data harmonization across networks.
  ○ Integrate remote sensing and other sensing technologies with in situ observations to improve spatial coverage and provide large‐scale model validation and constraints.
- Strategic model selection:
  ○ Ensure diversity in model structure and parameterizations, data sources, and calibration methods.
  ○ Avoid redundancy and over‐reliance on a group of models with overlapping codebases or parameterizations.
- Proper model execution and reporting:
  ○ Standardize evaluation software/platform, so that results can be published with transparency and be accessible to everyone interested.
  ○ Standardize data formatting, scenario definitions, and temporal/spatial resolutions to avoid discrepancies.
  ○ Run models across identical scenarios and boundary conditions for comparability.
  ○ Standardize MME outputs in terms of spatial–temporal resolutions, mean/variance of targeted variables from models, and other specific MME needs.
  ○ Document model versions, configurations, and known limitations to facilitate reproducibility and interpretation.
- Aggregation and communication:
  ○ Choose aggregation methods based on the MIPs and stakeholder needs.
  ○ Tailor designs of scenarios and outputs to address stakeholder/user needs (e.g., probabilistic forecasts for policymakers vs. mechanistic insights for researchers).
  ○ Clearly communicate sources of uncertainty, confidence levels, assumptions underlying projections, and limitations.
  ○ Provide contextual interpretation of results to support actionable decision‐making.

### 5.2 Best Practice 2: MIPs as Platforms for Model Improvement and Scientific Advancement

A critical but sometimes overlooked role of MIPs is to serve as iterative platforms for model improvement and scientific advancement. While this role has been performed in some CMIP and AgMIP activities (Asseng et al. 2013; Eyring et al. 2016), iterative model improvement through MIPs is still limited for ecosystem models, and many MIPs workflows often lack built‐in mechanisms to feed intercomparison results directly back into model development (Fer et al. 2021; Hassler et al. 2026; Rynne et al. 2025). MIPs should provide a coordinated mechanism to identify where models diverge, why those divergences occur, and what specific processes or parameterizations require refinement (Asseng et al. 2013; Eyring et al. 2016; Huntzinger et al. 2013; Wang et al. 2017; Figure 1). Box 4 provides a flowchart to improve a specific targeted process for model improvements as part of the MIP effort. This iterative cycle of evaluation and improvement ensures that models evolve based on both diagnostic insights and emerging empirical data, rather than remaining static or siloed within individual research groups.

BOX 4 **How to iteratively improve models through MIPs?**

- Step 1: Identify targets
  ○ Pinpoint the scientific gaps (specific processes, input terms, or targeted variables) that drive model uncertainty.
  ○ Focus on specific intermediate processes (e.g., plant respiration, litter decomposition) rather than highly aggregated outcomes (e.g., crop yield or soil organic carbon) that combine multiple interactions.
  ○ Clearly define the scientific questions and prioritize key uncertainty drivers that limit model accuracy.
- Step 2: Secure benchmark data
  ○ Collect diverse, high‐quality observational data that ideally represent a comprehensive range of climate, soil, and management variability.
  ○ Ensure data are suitable for process‐level evaluation, and where gaps exist, identify opportunities for new experiments or measurements to strengthen benchmarking data.
- Step 3: Formulate executable protocols
  ○ Develop transparent, modular MIP protocols with well‐defined input levels, scenarios, and data constraints.
  ○ Establish standardized evaluation metrics to support both process‐level and system‐level diagnostics, enabling consistent and reproducible model intercomparisons.
- Step 4: Run the protocol
  ○ Conduct MIP experiments following the standardized procedures across models and phases.
  ○ Use observations to constrain parameters through model‐data fusion techniques to reduce individual models' biases.
  ○ Perform objective benchmarking of process‐level governing equations and system‐level outputs.
  ○ Isolate key parameterizations or mechanisms to understand model behavior and performance differences.
- Step 5: Review and discover gaps
  ○ Evaluate results to identify processes with large inter‐model variability, systematic biases, or poor agreement with observations.
  ○ Diagnose data and knowledge gaps that constrain those processes, and propose targeted model refinements or new data collection efforts.
- Step 6: Repeat and iterate
  ○ Propose and incorporate improvements, such as refined parameters or updated process representations, into individual models, while carefully considering the underlying structural assumptions and interactions among model components.
  ○ Re‐run standardized MIP experiments to compare results, quantify uncertainty reduction, and demonstrate progressive model improvement through iterative learning cycles.

To facilitate this evolution, MIPs should promote shared code repositories, version tracking, modular design, and open documentation, fostering transparency and collaboration across the modeling community (Collier et al. 2018; Easterbrook 2014; Eyring et al. 2016; Huntzinger et al. 2013; Wang et al. 2024). This approach aligns with the principles of open science, encouraging community‐driven innovation and reducing redundancy in model development. Over time, such collaboration accelerates the convergence toward improved, co‐developed models that better capture system dynamics and uncertainties, ultimately strengthening both individual models and the MMEs framework that builds upon them.

We fully acknowledge that improving models through MIPs contains practical challenges that we need to overcome. Individual models are often built upon fundamental structural assumptions and process representations that define their behavior and scope of application. These assumptions typically involve tightly coupled components (e.g., canopy structure, leaf traits, and photosynthesis or respiration processes), meaning that modifying one element may introduce unintended consequences elsewhere in the model. As a result, model improvements should also be pursued carefullly through systematic, hypothesis‐driven refinements of parameterizations and process representations, guided by observational data, cross‐scale validation, and explicit evaluation of underlying mechanisms.

### 5.3 Best Practice 3: Engage Stakeholders Early and Throughout the MME Process

For MMEs to be useful and actionable, they must address real‐world questions or problems faced by practitioners, whether they are policymakers, land managers, or private sector actors. These stakeholders often operate under tight timelines and specific constraints, requiring insights that are not only scientifically robust but also timely, interpretable, and directly relevant to decision‐making contexts. To meet these needs, stakeholder engagement must begin early in the modeling process and continue throughout the entire MME development cycle (Figure 1), a critical aspect that has often been overlooked in previous studies.

*Stakeholder co‐design* plays a vital role in ensuring that MME outputs align with user needs (Dingley 2023; Rosenzweig and Hillel 2015). This collaborative approach allows practitioners and scientists to frame relevant policy scenarios, define acceptable uncertainty thresholds, and interpret results in ways that are both actionable and context‐specific. For example, rather than presenting point estimates, many MMEs could deliver probabilistic ranges (e.g., “this MME has ±20% confidence”), accompanied by clear explanations of the sources and implications of uncertainty. Practitioners should be encouraged to demand uncertainty quantifications as a standard component of MME outputs. Incorporating risk analysis into this process would allow stakeholders to further assess the likelihood and consequences of different outcomes, linking model outputs directly to decision thresholds and vulnerability assessments.

At the same time, scientists have a responsibility to proactively engage with stakeholders, ensuring that models remain relevant and responsive to societal needs. This requires overcoming the academic tendency to only prioritize methodological perfection over practical utility and to recognize that stakeholders often need timely responses, which sometimes represent approximate insights within a short timeframe, rather than delayed, overly precise answers. Examples of successful co‐design include the use of CMIP for climate policy design (Dingley 2023) and crop adaptation in AgMIP (Rosenzweig and Hillel 2015; Bartels et al. 2017). These partnerships demonstrate how early engagement between scientists and practitioners can shape model assumptions, scenario design, and uncertainty communication, ensuring that MME outputs are not only scientifically credible but also directly usable in policy and management contexts.

## 6 Conclusion and Perspectives: Building a Credible MME Paradigm for Ecosystem Modeling

MMEs have the potential to transform ecosystem modeling into a powerful tool for environmental policy design, sustainability planning and investment, and decision‐making. Yet they are not plug‐and‐play solutions. We should first learn from the past successes and lessons of existing MIPs‐MMEs efforts, such as CMIP and AgMIP. The value of MMEs depends closely on the rigor of MIPs that underpin them and the stakeholder engagement that shapes them. Without robust diagnostics from MIPs, MMEs risk offering false confidence; without practitioner involvement, they risk irrelevance. Seen within an Expectation–Maximization paradigm, the long‐term credibility of MMEs emerges from their continual grounding in evaluation, learning, and feedback rather than from ensemble model size alone. To realize the full potential of MMEs, the ecosystem modeling community must invest in shared infrastructure and long‐term collaboration, and take advantage of AI when necessary. This effort includes the development of standardized protocols for both MIPs and MMEs, ensuring consistency and transparency in model evaluation and ensemble construction. Shared, open‐access benchmark datasets are equally vital: these datasets must represent the geographic, climatic, and ecological diversity of real‐world systems across a wide range of spatial and temporal scales. It is also critical to treat MIPs‐MMEs not as static endpoints but as an iterative, co‐evolving platform for advancing the scientific process and innovation, especially the inclusion of effective model improvement, to advance both scientific understanding and overall modeling capability. Policy and funding bodies play a crucial role in supporting this vision. Investment is needed to sustain benchmark data collection, support MIP coordination hubs, and build communities of practice that democratize MIP and MME protocols/methodologies as well as further model improvement. Resources should also be directed toward fostering structured scientist‐stakeholder partnerships and open‐source platforms for reproducible ensemble workflows, enabling broader participation and transparency. In addition, academic communities (e.g., journals and peer reviewers) should raise expectations for methodological rigor, documentation, and reporting transparency in MME studies, ensuring that ensemble outputs are credible and decision‐relevant. By investing in these foundational elements, the scientific community can elevate MMEs from ad hoc aggregation of models into a trusted backbone of environmental policy decision‐making.

## Author Contributions

**Kaiyu Guan:** conceptualization, formal analysis, funding acquisition, investigation, methodology, resources, supervision, writing – original draft, writing – review and editing. **Rongzhu Qin:** conceptualization, formal analysis, investigation, methodology, resources, writing – original draft, writing – review and editing. **Zewei Ma:** formal analysis, investigation, visualization, writing – original draft, writing – review and editing. **Lei Zhao:** investigation, writing – review and editing. **Licheng Liu:** investigation, writing – review and editing. **Sheng Wang:** investigation, writing – review and editing. **Trevor F. Keenan:** funding acquisition, writing – review and editing. **Mengqi Jia:** writing – review and editing. **Debjani Sihi:** funding acquisition, writing – review and editing. **Eric Potash:** writing – review and editing. **Senthold Asseng:** writing – review and editing. **Jingyun Tang:** funding acquisition, writing – review and editing. **Xiangtao Xu:** writing – review and editing. **Katheleen Boomer:** writing – review and editing. **Bin Peng:** funding acquisition, writing – review and editing. **Zhenong Jin:** writing – review and editing. **Forrest M. Hoffman:** funding acquisition, writing – review and editing. **Stephen Ogle:** funding acquisition, writing – review and editing. **Jonathan Coppess:** writing – review and editing. **Madhu Khanna:** writing – review and editing. **Alyssa Whitcraft:** writing – review and editing. **Benjamin R. K. Runkle:** funding acquisition, writing – review and editing.

## Conflicts of Interest

The authors declare no conflicts of interest.

## Data Availability

The authors have nothing to report.

## Appendix A

Here, we first provide a general derivation for errors measured by the mean square error (MSE) in MMEs. Then, we prove that the case in Box 2 is a simplified case where the correlation between every two models is the same. Let $f\left(x\right)$ denote the true function of the ecosystem, where x represents inputs such as precipitation or temperature. An ensemble consists of I models, where the prediction of the i‐th model for the m‐th sample is $\hat{f_{i}}\left(x_{m}\right)$. The fundamental error of any single model prediction, $\varepsilon_{i,m}$, is the deviation from the truth:

(A1)
$$\varepsilon_{i,m}={\hat{f}}_{i}\left(x_{m}\right)-f\left(x_{m}\right)$$

The error terms can then be decomposed into two parts. One is the bias, $\beta_{i,m}$, which represents the systematic deviation from the true value. Another is the variance, $s_{i,m}$, which characterizes the stochastic error,

(A2)
$$\varepsilon_{i,m}=\left(E\left[{\hat{f}}_{i}\left(x_{m}\right)\right]-f\left(x_{m}\right)\right)+\left({\hat{f}}_{i}\left(x_{m}\right)-E\left[{\hat{f}}_{i}\left(x_{m}\right)\right]\;\right)$$

(A3)
$$\varepsilon_{i,m}=\beta_{i,m}+s_{i,m}$$

(A4)
$$\beta_{i,m}=E\left[{\hat{f}}_{i}\left(x_{m}\right)\right]-f\left(x_{m}\right)$$

(A5)
$$s_{i,m}={\hat{f}}_{i}\left(x_{m}\right)-E\left[{\hat{f}}_{i}\left(x_{m}\right)\right]$$

Thus, the error of the ensemble at the m‐th sample is,

(A6)
$$\varepsilon_{\mathrm{ens}}=\frac{1}{I}\sum_{i=1}^{I}\left(\beta_{i,m}+s_{i,m}\right)$$

The MSE of the ensemble model is the expected value of the squared error,

(A7)
$$\mathrm{MS}E_{\mathrm{ens}}=E_{m}\left[{\left(\varepsilon_{\mathrm{ens},m}\right)}^{2}\right]=E_{m}\left[{\left(\frac{1}{I}\sum_{i=1}^{I}\left(\beta_{i,m}+s_{i,m}\right)\right)}^{2}\right]$$

(A8)
$$\mathrm{MS}E_{\mathrm{ens}}={\left(\frac{1}{I}\sum_{i=1}^{I}\beta_{i}\right)}^{2}+\mathrm{Var}\left(\frac{1}{I}\sum_{i=1}^{I}s_{i}\right)$$

(A9)
$$\mathrm{MS}E_{\mathrm{ens}}={\left(\frac{1}{I}\sum_{i=1}^{I}\beta_{i}\right)}^{2}+\frac{1}{I^{2}}\sum_{i=1}^{I}\sum_{j=1}^{I}\mathrm{Cov}\left(s_{i},s_{j}\right)$$

(A10)
$$\begin{array}{c} \mathrm{MS}E_{\mathrm{ens}}={\left(\frac{1}{I}\sum_{i=1}^{I}\beta_{i}\right)}^{2}+\frac{1}{I^{2}}\left(\sum_{i=1}^{I}{\sigma_{i}}^{2}+\sum_{i\neq j}\rho_{i,j}\sigma_{i}\sigma_{j}\right) \end{array}$$

where $\mathrm{Var}\left(\cdot \right)$ is the variance; $\mathrm{Cov}\left(\cdot ,\cdot \right)$ is the variance; $\rho_{i,j}$ is the correlation between the stochastic errors in the i‐th model, $s_{i}$, and the j‐th model, $s_{j}$; $\sigma_{i}$ is the variance of the stochastic errors in the i‐th model.

If the correlation between every two models is the same, is:

(A11)
$$\rho_{i,j}=\rho ,\text{if}\;i\neq j$$

(A12)
$$\rho_{i,j}=1,\text{if}\;i=j$$

Then,

(A13)
$$\mathrm{MS}E_{\mathrm{ens}}={\left(\frac{1}{I}\sum_{i=1}^{I}\beta_{i}\right)}^{2}+\frac{1}{I^{2}}\left(\sum_{i=1}^{I}\sigma_{i}^{2}+\sum_{i\neq j}\rho \sigma_{i}\sigma_{j}\right)$$

(A14)

(A15)
$$\mathrm{MS}E_{\mathrm{ens}}={\left(\frac{1}{I}\sum_{i=1}^{I}\beta_{i}\right)}^{2}+\frac{1-\rho }{I^{2}}\sum_{i=1}^{I}\sigma_{i}^{2}+\rho {\left(\frac{1}{I}\sum_{i=1}^{I}\sigma_{i}\right)}^{2}$$

where Equation (A15) is exactly the Equation (9) in Box 2.

## Appendix B

To illustrate the effectiveness of ensemble models, we conducted a series of numerical experiments: (1) A baseline scenario, designed to demonstrate the performance of ensemble averaging and the model selection strategy. (2) Bias experiments, designed to assess how systematic model bias affects ensemble performance and to evaluate the impact of model inter‐dependence. (3) Dependence experiments, designed to examine how varying inter‐model correlation influences the effectiveness of ensemble models. The model performance was assessed with root mean square error (RMSE).

- Baseline setup: To mimic the model ensemble process, we generated 50 models with the two additional assumptions following Equation (7). Each model gave 100 predictions.
  1. The bias, $\beta_{i}$, follows a standard normal distribution $\beta \sim N\left(\mu_{\beta }=0,{\sigma_{\beta }}^{2}=1\right)$.
  2. The model noise level, $\sigma_{i}$, follows a uniform distribution, $\sigma_{i}\sim U\left[a=0,b=1\right].$
  3. ρ=0, which represents no dependence in all 50 models.
- Experiment setup for different biases: To investigate the effect of systematic bias, we constructed three experimental groups by modifying the baseline setup with different bias magnitudes. Specifically, $\mu_{\beta }=0.2,0.4,0.6$. All other settings remained identical to the baseline experiment.
- Experiment setup for different model inter‐dependence: To investigate the effect of systematic bias, we constructed three experimental groups by modifying the baseline setup with different correlation coefficients. Specifically, ρ=0.25,0.50,0.75. Other settings are the same as baseline.

Notably, with the assumptions that the bias follows the normal distribution and the noise level follows a uniform distribution, the expectation of the MSE can be given by,

(B1)
